# North American Prairie Is a Source of Pollen for Managed Honey Bees (Hymenoptera: Apidae)

**DOI:** 10.1093/jisesa/ieab001

**Published:** 2021-02-23

**Authors:** Ge Zhang, Ashley L St. Clair, Adam G Dolezal, Amy L Toth, Matthew E O’Neal

**Affiliations:** 1 Department of Entomology, Iowa State University, Ames, IA, USA; 2 Department of Ecology, Evolution and Organismal Biology, Iowa State University, Ames, IA, USA; 3 Department of Entomology, University of Illinois Urbana-Champaign, Urbana, IL, USA

**Keywords:** tallgrass prairie, habitat, beekeeping, foraging preference, landscape

## Abstract

Prairie was a dominant habitat within large portions of North America before European settlement. Conversion of prairies to farmland resulted in the loss of a large proportion of native floral resources, contributing to the decline of native pollinator populations. Efforts to reconstruct prairie could provide honey bees (*Apis mellifera*) a source of much-needed forage, especially in regions dominated by crop production. To what extent honey bees, which were introduced to North America by European settlers, use plants native to prairies is unclear. We placed colonies with pollen traps within reconstructed prairies in central Iowa to determine which and how much pollen is collected from prairie plants. Honey bee colonies collected more pollen from nonnative than native plants during June and July. During August and September, honey bee colonies collected more pollen from plants native to prairies. Our results suggest that honey bees’ use of native prairie plants may depend upon the seasonality of both native and nonnative plants present in the landscape. This finding may be useful for addressing the nutritional health of honey bees, as colonies in this region frequently suffer from a dearth of forage contributing to colony declines during August and September when crops and weedy plants cease blooming. These results suggest that prairie can be a significant source of forage for honey bees in the later part of the growing season in the Midwestern United States; we discuss this insight in the context of honey bee health and biodiversity conservation.

The tallgrass prairie biome of the North American Great Plains consists of many native plants that can support a diverse pollinator community (Tonietto et al. 2011, Smith et al. 2012, Robson 2014, Coleman 2019, Lamke 2019). The honey bee, *Apis mellifera*, was introduced to North America by European settlers and is a highly generalist forager known to take advantage of a wide variety of wild and cultivated flowering plant species. Foraging honey bees have been observed visiting many native plants found in prairies (Tuell et al. 2014, Carr-Markell et al. 2020). Although there are few hectares of prairie left due to widespread land conversion for urban and agricultural use in the Midwestern United States, some beekeepers target prairie or locations near prairie to install their apiaries (Werling et al. 2014, Otto et al. 2018) for the production of a honey crop (Nelson and Jay 1982).

Honey bees have also been observed collecting pollen from plants in prairies (Carr-Markell et al. 2020), however, the abundance and diversity of pollen collected across the growing season is not well studied when honey bee colonies are located in prairies. Although pollen is only a minor contributor to the weight of a colony, pollen is an important source of essential nutrients for colony and individual honey bee growth (Wright et al. 2018). Determining which species of plants within prairies are a source of pollen and how much pollen is collected from prairies across the season could provide insight into how best to select native plants for habitat restoration that benefit both honey bees and native bees.

In addition to plants from habitats that are immediately adjacent to an apiary, honey bees also forage on plants widely distributed within the surrounding landscape, commonly within 10 km from their colonies (Beekman and Ratnieks 2000). Thus, for colonies located near or within small islands of prairies in an agricultural or urban matrix, both native and nonnative plants have the potential to be a source of forage. Honey bees in North America have access to many nonnative plants earlier in the season, such as white clover (*Trifolium repens* L. [Fabales: Fabaceae]) (Sponsler et al. 2017), which is commonly used as forage in both their native (Europe) and nonnative (North America) ranges. Honey bee colonies in agricultural landscapes dominated by soybean and corn do not experience early season forage shortage but suffer from late-season decline in colony weight after crops and clovers senesce (Dolezal et al. 2019). However, colonies that are moved to prairies can avoid the late-season colony weight loss and even gain weight, suggesting prairie may be a valuable forage resource. Because honey is the heaviest component of colony weight, this weight gain suggests that prairie is a source of nectar. To what extent honey bees use plants in prairies as pollen sources in North America is unclear, especially when they are living in a wider landscape with nonnative resources present.

In the current study, we contribute to the knowledge base of honey bees’ usage of North American native prairie plants. Specifically, we consider the phenology of how honey bees use native prairie plants and nonnative plants for pollen across a growing season by placing apiaries in reconstructed prairies in central Iowa, United States. Iowa’s landscape contains small islands of prairie embedded within a matrix of farmland that is primarily committed to the production of corn and soybean (USDA-NASS 2019). We hypothesized that honey bees in a mixed crop/natural prairie landscape would forage on a diverse combination of both nonnative and native plants. We focused on bees placed in reconstructed prairies within a corn–soybean crop dominated landscape and predicted that honey bee colonies placed in prairie habitat would use plants found within prairies as a source of pollen throughout the growing season. However, because many nonnative weedy plants favored by bees (e.g., clovers) cease blooming later in the season, we also predicted proportionally more pollen would come from native prairie plants later in the season compared to earlier in the season. We also explored the potential correlation of pollen collection with the extent of agricultural land cover in the surrounding landscape. The results of this study can provide useful insights into the utility of prairies for forage by the beekeeping industry, as well as inform conservation management decisions related to specific prairie plants and time periods presenting potential forage competition with wild bees.

## Materials and Methods

### Prairies and Land Cover of Surrounding Landscapes

**Fig. 1.**
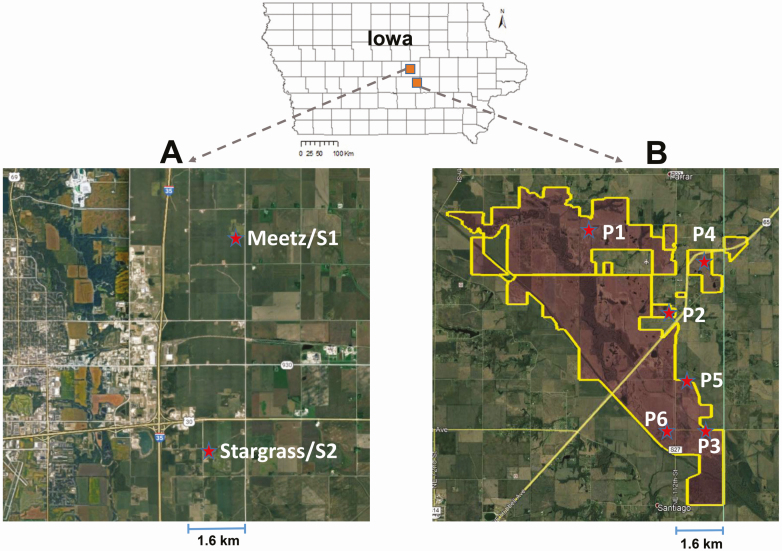
Location of apiaries. The apiaries are marked with red star-shaped symbols. A) Apiaries (S1 and S2) were installed at Meetz and Stargrass prairies in Story County in both 2016 and 2017. B) Six apiaries (P1–P3 in 2016 and P4–P6 in 2018) were installed at different prairies at Chichaqua Bottoms Greenbelt Conservation Area (highlighted by yellow boarder in the map) in Polk County. The outline of Chichaqua Bottoms Greenbelt Conservation Area was provided by Doug Sheeley, Polk County (Iowa) Conservation. The other information about those apiaries and prairies was summarized at Table 1.

We used two types of reconstructed tallgrass prairies located in Iowa; isolated, reconstructed tallgrass prairie and integrated, reconstructed tallgrass prairie based on the presence of other prairies adjacent to the focus prairie, as described in previous studies (Shepherd and Debinski 2005, Orlofske et al. 2011). In our study, isolated reconstructed prairies did not have other prairies near them, but integrated reconstructed tallgrass prairies did. In Story County, we used two isolated reconstructed tallgrass prairies (named Meetz and Stargrass) to install our apiaries (S1 and S2) during both 2016 and 2017 (Fig. 1, Table 1). In a conservation area, Chichaqua Bottoms Greenbelt in Polk County in central Iowa, we used three integrated, reconstructed-tallgrass prairies for our apiaries during 2016 (P1, P2, and P3) and another three integrated, reconstructed-tallgrass prairies for three apiaries during 2018 (P4, P5, and P6, Fig. 1, Table 1). The integrated reconstructed prairies were larger than the isolated reconstructed prairies. These prairies were not mowed during the study period. One apiary was installed at each prairie during 2016–2018 resulting in five, two, and three replications of apiaries in 2016, 2017, and 2018, respectively. Because we used two prairies (Meetz and Stargrass) in two continuous years (2016 and 2017), a total of eight prairies were used in this study.

**Table 1.** Summary of information about prairies and apiaries in the current study

Year	Prairie name	Prairie type^b^	County	Hectare	Apiary symbol	Apiary Latitude	Apiary Longitude	Colonies per apiary	Pollen traps per apiary
2016	Meetz	ISO	Story	15	S1	42.059197	−93.541481	1	1
2016	Stargrass	ISO	Story	10.42	S2	41.999103	−93.554372	1	1
2016	Darnell-Holy Cross (CBG)^a^	INT	Polk	20.23	P1	41.791219	−93.401356	1	1
2016	Bailey-Carpenter (CBG)	INT	Polk	47.75	P2	41.759444	−93.373889	1	1
2016	Barrer (CBG)	INT	Polk	31.57	P3	41.731672	−93.352764	1	1
2017	Meetz^b^	ISO	Story	15	S1	42.056628	−93.541527	2	2
2017	Stargrass^b^	ISO	Story	10.42	S2	41.999103	−93.554372	2	2
2018	Engeldinger Marsh (CBG)	INT	Polk	36.42	P4	41.776394	−93.34951	4	2
2018	Kunze (CBG)	INT	Polk	69.20	P5	41.746154	−93.365855	4	2
2018	Lloyd Bailey (CBG)	INT	Polk	42.49	P6	41.731685	−93.368204	4	2

^a^CBG, Chichaqua Bottoms Greenbelt Conservation Area in Polk County in Iowa, USA. Two sets of three prairies in 2016 and 2018 in CBG were selected for installing our apiaries, respectively. Any prairie selected in CBG will have other prairies nearby, thus we identified them as integrated prairies.

^b^We used the same prairies, i.e., Meetz and Stargrass, in both 2016 and 2017 for our apiary locations. ISO, isolated, reconstructed tallgrass prairie. INT, integrated, reconstructed tallgrass prairie.

**Fig. 2.**
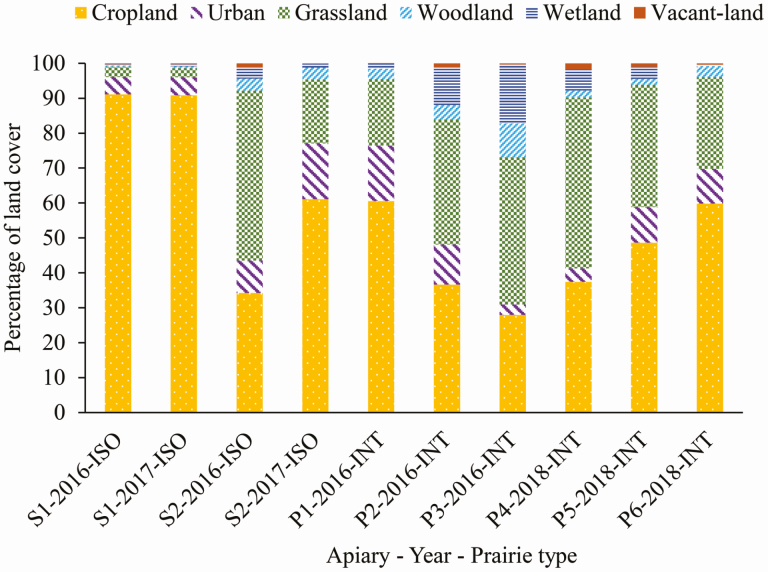
Percent of six general land cover type composing the landscapes around apiaries within a 1.6 km radius. ISO, isolated reconstructed tallgrass prairies, INT, integrated reconstructed tallgrass prairies.

To help account for the potential impact that the surrounding landscapes may have on honey bee pollen collection, the percent of land cover types was measured using ArcGIS (Esri, Redlands, CA). Although honey bees can forage up to 13.5 km away from their colony, most bees forage within a 1.6 km radius around the colony (Beekman and Ratnieks 2000, Carr-Markell et al. 2020), and the land cover within this buffer has been observed to influence honey bee health (Couvillon et al. 2014, Otto et al. 2016, Dolezal et al. 2019). Therefore, the percent of land cover types was measured within 1.6 km radius of each apiary. The land cover data layer was from USDA-NASS Cropscape (https://nassgeodata.gmu.edu/CropScape/). These data are updated every year and we used the land use surrounding each apiary in the corresponding year. In total, 21 land cover types were identified and grouped into six major types for this study, including cropland, urban, grassland, woodland, wetland, and vacant-land (Supp Table 1 [online only]). Cropland was the most common land cover in the landscapes surrounding apiaries located in prairies (Fig. 2). Grassland was the second most common land cover; this measure included the area of prairie where we installed apiaries (Fig. 2).

### Apiary Establishment and Management

All the colonies were derived from managed stocks of Italian bees, *Apis mellifera ligustica*, first located at the Iowa State University (ISU) Research Apiary at the Horticulture Research Station in Ames, Iowa, USA, on 6 May 2016, 2 May 2017, and 6 June 2018. Colonies were housed in standard-sized (‘deep’) Langstroth hive boxes with ten frames. Colonies used in 2016 and 2018 were initiated from ‘nucleus colonies’ that contained frames with adult bees, immature bees (eggs, larvae, and pupae), a honey bee queen, and drawn comb with honey. Colonies used in 2017 were initiated from ‘package bees’ (composed of adult bees and a honey bee queen held in wooden box with wire mesh made to conveniently deliver honey bees). To avoid early spring food shortage and make starting colonies similar between years, colonies initiated from packages bees in 2017 were provided with frames of drawn comb and honey. The only difference between colonies created by package and nucleus bees was the presence of brood (eggs, larvae, pupae) in the nucleus. The initial adult bee populations were similar across years (2016–2018) with approximately 7,000 adult bees per colony regardless of how they were initiated (packages vs nucleus colonies). The starting colony weights (including weight from bees, wax comb, honey) ranged between 5.41 to 9.91 kg in 2016, 6.52 to 8.79 kg in 2017, and 6.5 to 8.95 kg in 2018, when the wooden components (hive boxes, frames, bottom board, and lid) were excluded. After colonies grew to a sustainable size, no supplemental food was given to colonies.

All honey bee colonies were moved to prairies within three days after the colonies were created at the ISU apiary. The number of colonies in an apiary located in a prairie varied by year; one, two, and four colonies were included in each apiary during 2016, 2017, and 2018, respectively. We managed apiaries in prairies with a slightly different frequency of apiary inspection depending on year and season; once in May and twice per month from June to August in 2016; once in June and October and twice per month from July to August in 2017; once in June, September, and October, four times in July, twice in August in 2018. Any two inspections were separated by at least seven days. Each apiary inspection included a series of activities described as follows. Additional hive boxes were added if colonies lacked space to generate more brood or honey. Presence of the queen (i.e., visual confirmation of the queen, presence of eggs laid, or presence of young larvae less than three days old) was checked and a new mated queen was introduced within three days when the queen’s presence was not observed. We experienced queen losses of two, zero, and one in 2016, 2017, and 2018, respectively. To reduce infestations of Varroa mites (*Varroa destructor* [Mesostigmata: Varroidae]), colonies were treated with a miticide (Apilife Var; Chemicals Laif SPA, Vigonza, Spain) once in August and September during 2016, and twice in September during 2017. During 2018, colonies were treated with Apiguard (Vita Europe Ltd, Valdosta, USA) once in August and September.

### Pollen Collection and Identification

Pollen collected by honey bee foragers was harvested using hive entrance pollen traps (Brushy Mountain Bee Supply, Wilsonville, USA) placed on individual colonies. A plastic plate with star-shaped holes inserted into pollen traps pulled pollen pellets off the hind legs of foragers when they entered the hive. When pollen was not being collected, the plastic plate was removed from the trap so honey bees could leave and enter without being disturbed. The number of pollen traps at an apiary varied by year: one in 2016 and two in 2017 and 2018 (Table 1). Pollen was collected one to five times per month during a 24 h period without rainfall (frequency of collection summarized at Supp Table 2 [online only]). Across the season, we had 13, 5, and 7 pollen collections in 2016, 2017, and 2018 respectively. Due to variation in the number of apiaries and colonies within each apiary, those pollen collections resulted in 75, 16, and 21 samples collected in this three-year period. After removing nonpollen debris, all pollen collections were weighed and stored at −20°C.

To test our first prediction those colonies placed in prairies would continuously use plants found within prairies as a source of pollen, we used microscopy to identify the source of pollen collected by pollen traps. If an apiary had one pollen trap, 2 g pollen was extracted from the only pollen sample collected from that trap for that 24 h period. If an apiary had two pollen traps, half of the pollen from each trap was mixed, 2 g pollen was extracted from the mixture, and the pellets were sorted by color. Pellets of the same color were weighed again and mixed with Cablerla’s fluid with fuchsin dye. The pollen solution was pasted on a glass slide for taxonomic identification using a compound microscope. Morphological features of pollen were used to determine the plant species that produced this pollen. Pollen collected by honey bees was compared to a library of reference pollen created by extracting pollen from flowering plants collected adjacent to apiaries (the plant taxa in the library referred in Supp Table 3 [online only]). The flowering plants in the reference pollen library were collected within 15 m from apiaries placed at the prairies, as well as additional apiaries managed throughout Iowa as part of other experiments in 2015–2018 (Dolezal et al. 2019, Zhang et al. 2020). In total, 89 plant species were included in the pollen reference library, comprised of 49 native and 40 nonnative plants (Supp Table 3 [online only]). Because honey bees may forage widely to collect pollen (e.g., 314 km^2^ if they scout up to 10 km radius), it is possible that rare plants used by honey bees were not encountered in our survey of plants around the apiaries. This may explain why a subset of pollen types could not be matched to plants in our library, and pollen from those plants were thus not able to be identified by microscopy. Pollen collected by honey bees with morphological characteristics that did not correspond to specimens in the reference library was given a unique morphospecies identification (Supp Table 4 [online only]). Percent of pollen from a plant taxon was calculated by dividing the weight of pollen pellets from each color by the 2 g sample mass.

### Statistical Analysis

To test our first prediction about the effect of seasonality on bee pollen collection, we compared plant pollen taxa (richness) and pollen abundance among months. An apiary installed at a prairie was an experimental unit. Because the frequency at which pollen was collected from the hive varied from one to five times per month (Supp Table 2 [online only]), a date within a month was selected that was as similar as possible to dates in the same month for each year (Supp Table 2 [online only]). Taxon richness of pollen collected per apiary was compared among different months using ANOVA within JMP 15 Pro (SAS Institute, Cary, NC) as data were normally distributed (Shapiro–Wilk test W = 0.96, *P* = 0.12). Abundance of pollen (g) was compared among months using Wilcoxon test as data were not normally distributed (Shapiro–Wilk test W = 0.58, *P* < 0.0001).

To test our second prediction that colonies would collect more pollen from native plants later in the season as floral resources decline in agricultural landscapes, we grouped pollen sources into two categories, i.e., native versus nonnative. We defined a plant as ‘native’ if it is considered a component of prairies and was not introduced to North America. We defined a plant as ‘nonnative’ if it was introduced to North America or if it is not considered a component of North American prairies, e.g., a weedy species. The designation of a plant to these two categories (native vs nonnative) was based on the Natural Resource Conservation Service (NRCS), United States Department of Agriculture (USDA) (https://plants.sc.egov.usda.gov/java/). Number of plant taxa was counted for each category for estimating taxon richness. Percent of pollen by mass was used as an indicator of relative abundance of pollen collected by honey bees either from native or nonnative plants. We compared the plant taxon richness or percent of pollen derived from native and nonnative plants using two separate linear mixed effect models within the PROC Mixed function (SAS Institute, Cary NC). Data were normally distributed and data from all collection dates were included in the statistical analysis. Response variables included plant taxon richness or percent of pollen collected, and pollen category was an explanatory variable. In this mixed effect model, we also included the interaction of pollen categories (i.e., native or nonnative) with year or prairie type. To test the robustness of our results given the large number of unidentified morphospecies, we used three different hypothetical group assignment parameters, 100% the unidentified morphospecies to nonnative, 100% to native, or 52% to nonnative and 48% to native, the latter reflecting the percentage from identified pollen. We then re-analyzed the data on native versus nonnative taxa composition with the same mixed effect model described above.

To explore if land cover type in the surrounding landscapes explained the variation in taxon richness and abundance of pollen, we conducted a linear regression analysis of plant taxon richness and percent of pollen derived from native and nonnative plants. The response variables were plant taxon richness and percent of pollen derived from native and nonnative plants, and the six land cover types were explanatory variables. We used a stepwise model selection to determine which land cover was most highly correlated with taxon richness and relative abundance of pollen derived from native or nonnative plants. Any land cover type meeting a 0.15 significance level was included in the model for further selection; while land cover categories not meeting a 0.15 significance level were removed from the model selection process (Littell et al. 2002). The regression analysis was conducted for each month separately.

## Results

### Honey Bees Use Several Plants Found in Prairie as Sources of Pollen

In total, 57 plant taxa were found in bee-collected pollen traps, across three years and eight prairies. This community was composed of 12 native plants, 13 nonnative plants, and 32 plants which were assigned a morphospecies designation (Table 2, Supp Table 4 [online only]). Although ‘unidentified morphospecies’ was the most numerous category, this pollen represented only 15 (±3)% of the average mass of pollen collected throughout the sampling periods (Table 2). For a given monthly time period including June through September across the three years of the experiment, the average number of morphospecies was 4.75 (±1), and the average number of identified species was 9.2 (±0.8), from June to September of 2016–2018.

**Table 2.** Average (±SE) percent of pollen derived from native and nonnative plants in each month of 2016–2018

Pollen taxa	2016 (*n* = 5)				2017 (*n* =2)				018 (*n* = 3)			
	June	July	Aug.	Sept.	June	July	Aug.	Sept.	June	July	Aug.	Sept.
Chamaecrista fasciculata*	0	10.01 ± 9.97	69.94 ± 17.76	0	0	0	79.94 ± 17.73	0	0	0.75 ± 0.75	61.34 ± 7.74	19.28 ± 15.81
Dalea purpurea*	0.02 ± 0.02	2.38 ± 1.70	0	0	0	30.61 ± 30.32	0	0	4.25 ± 4.25	0.56 ± 0.41	0	0
*Echinacea* spp.*	0	0	0	0	0	0	0	0	0	0.07 ± 0.07	0	0
Eryngium yuccifolium*	0	0	0	0	0	0	0	0	0	0.45 ± 0.45	0	0
*Helianthus, Heliopsis & Silphium* spp.*	0	0	0.47 ± 0.43	0	0.71 ± 0.71	1.28 ± 1.28	0.37 ± 0.37	12.80 ± 8.63	0	9.00 ± 8.77	0	0.62 ± 0.62
Iris versicolor *	25.01 ± 6.17	0.04 ± 0.04	0	3.82 ± 2.12	0	0	0	0	34.00 ± 8.11	0.42 ± 0.42	0	0
Monarda fistulosa & Pycnanthemum virginianum*	0	0.22 ± 0.22	0.05 ± 0.05	0	0	2.02 ± 2.02	0.26 ± 0.15	0.09 ± 0.09	0	2.47 ± 2.36	0	0
Oenothera biennis*	0	0	0	0	0	0	0	0	0	0.07 ± 0.07	0.64 ± 0.64	0
*Phlox* spp.*	0	0	0	6.40 ± 4.60	0	2.78 ± 2.78	0	0.07 ± 0.07	0	0	0.99 ± 0.15	0
Sambucus Canadensis*	0.02 ± 0.02	0	0	0	12.76 ± 5.72	0.54 ± 0.54	0	0	0.97 ± 0.35	0	0	0
*Solidago* spp.*	0	0	0.06 ± 0.05	26.92 ± 14.21	0	0	0.38 ± 0.38	80.58 ± 12.12	0	0	1.11 ± 1.11	36.15 ± 20.37
Tilia Americana*	0	0	0	0	0	0	0	0	1.33 ± 1.33	0	0	0
*Ambrosia* spp.* ^,§^	0	0	0.0 5 ± 0.04	0	0	0	10.56 ± 10.56	0.09 ± 0.09	0	0	0	0
Chenopodium album* ^,§^	0	0	0	0	0	0	0	0	0	0	1.31 ± 0.66	1.45 ± 0.76
*Cichorium intybus* ^§^	0	0	0	0	0	0	0	0	0	0.28 ± 0.28	0	0
*Cirsium* spp. ^§^	0.04 ± 0.02	0.09 ± 0.06	0.36 ± 0.27	0	6.63 ± 5.05	4.46 ± 4.4	0.41 ± 0.12	0	4.18 ± 3.17	0	0	0
*Daucus carota* ^§^	0	0	0	0	0	0	0	0	0	1.39 ± 1.39	0	0
*Lotus corniculatus* ^§^	6.25 ± 2.54	2.32 ± 1.43	0.4 ± 0.23	0	17.79 ± 17.79	0.66 ± 0.66	0	0	4.37 ± 2.19	0.47 ± 0.40	0.98 ± 0.98	0
*Melilotus* spp. ^§^	2.85 ± 1.41	0.51 ± 0.21	0.2 5 ± 0.25	0	0	0	0.10 ± 0.10	1.22 ± 1.22	29.46 ± 10.08	4.64 ± 1.98	0	0
*Pastinaca sativa* ^§^	0	0.44 ± 0.24	0.10 ± 0.10	0	6.45 ± 6.45	0	0	0	0	0	0	0
*Phaseolus vulgaris*	0.01 ± 0.01	0	0	0	0	0	0	0	0	0	0	0
*Taraxacum officinale* ^§^	0	0	0	3.64 ± 3.60	0	0.12 ± 0.12	0.47 ± 0.47	1.32 ± 0.33	0	0	0	0
*Trifolium pratense*	10.2 ± 7.79	52.3 ± 12.59	12.96 ± 8.02	24.58 ± 17.40	1.24 ± 1.24	8.08 ± 6.97	6.78 ± 6.34	0	0	2.91 ± 2.54	0	0.74 ± 0.74
*Trifolium repens*	32.63 ± 7.28	30.63 ± 13.47	14.45 ± 13.97	31.69 ± 19.21	54.42 ± 0.04	3.41 ± 2.89	0.09 ± 0.09	1.50 ± 0.23	13.89 ± 0.91	7.32 ± 4.48	2.46 ± 2.13	0
*Zea mays*	0.03 ± 0.03	0.02 ± 0.02	0	0	0	0	0.17 ± 0.17	0	0	46.72 ± 9.41	0	0
Total of unidentified pollen taxa ^a^	22.93 ± 6.39	1.02 ± 0.63	0.89 ± 0.56	2.93 ± 1.29	0	46.05 ± 44.64	0.46 ± 0.46	2.34 ± 2.23	7.55 ± 2.46	22.45 ± 14.44	31.16 ± 5.41	41.76 ± 26.45

*Native plants as pollen source. ^§^Invasive plants as pollen source. ^a^Each unidentified pollen taxa was record separately and designated by a morphospecies name (refer to Supp Table 4 [online only]).

The most common native plants represented in our pollen traps (>10 % by weight during any month across three years) were northern blue flag (*Iris versicolor* L. [Asparagales: Iridaceae]), purple prairie clover (*Dalea purpurea* Vent. [Fabales: Fabaceae]), common elderberry (*Sambucus Canadensis* L. [Dipsacales: Adoxaceae]), partridge pea (*Chamaecrista fasciculata* [Michx.] Greene [Fabales: Fabaceae]), goldenrod (*Solidago* spp. [Asterales: Asteraceae]), and sunflower (*Helianthus, Heliopsis & Silphium* spp. [Asterales: Asteraceae]). The most common identified nonnative plants represented in our pollen trap were white clover (*Trifolium repens* L. [Fabales: Fabaceae]), red clover (*Trifolium pratense* L. [Fabales: Fabaceae]), sweet clover (*Melilotus* spp. [Fabales: Fabaceae]), birdsfoot trefoil (*Lotus corniculatu*s L. [Fabales: Fabaceae]), and ragweed (*Ambrosia* spp. [Asterales: Asteraceae]) (Table 2). Despite being a common part of the central Iowa landscape, corn (*Zea mays* L. [Poales: Poaceae]) was only found in significant amounts during one month period (July of 2018) of this three-year study (Table 2). Soybean pollen was not present in the traps, which is consistent with a similar study conducted in central Iowa (Dolezal et al. 2019, Zhang et al. 2020).

### Plant Species Used for Pollen Varied by Plant Category Across the Growing Season

**Fig. 3.**
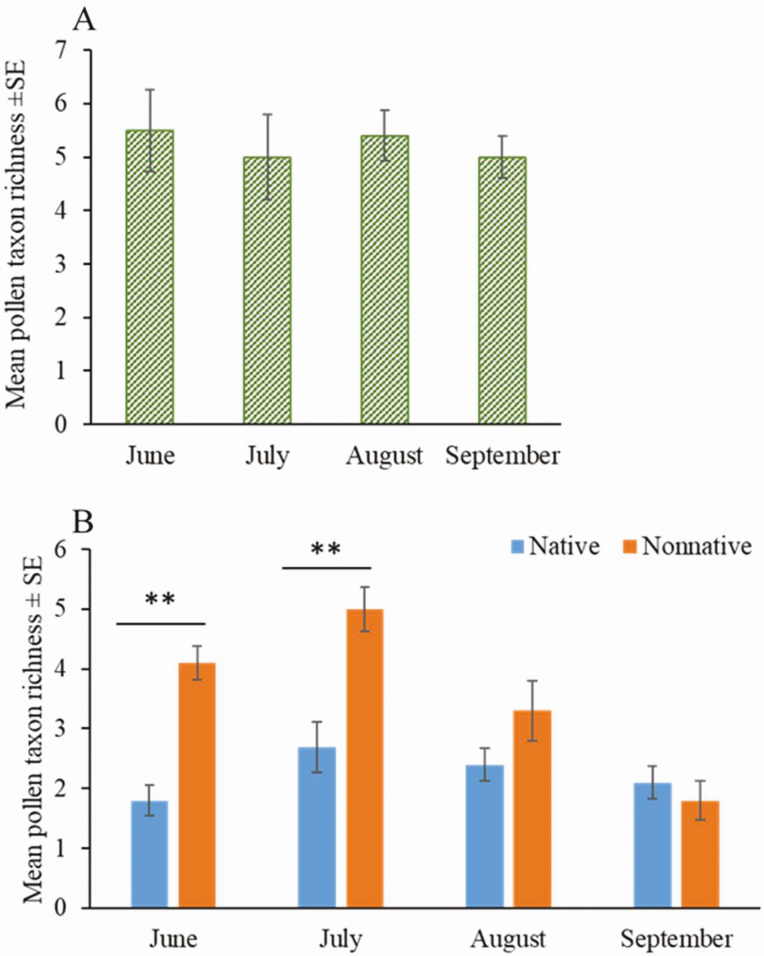
Taxon richness of pollen across the growing season (A) and comparison of taxon richness of pollen derived from native versus nonnative plants (B). Bars on each column represented the standard error. A) One pollen collection date in each month was selected for comparing the taxon richness of pollen across months (see Supp Table 2 [online only] for date selection). Taxon richness of pollen was not significantly different among months (*F* = 0.17; df = 3, 36; *P* = 0.9147). B) Data from all the pollen collection dates were used for comparing taxon richness of pollen derived from native versus nonnative. We found in June and July, percent of pollen from nonnative plants was significantly higher than that from native plants (** *P* < 0.01).

Bees collected a relatively constant number of plant taxa as pollen sources over the growing season. We found taxon richness of plants used by honey bee did not differ across months (*F* = 0.17; df = 3, 36; *P* = 0.9147), ranging from an average of five to six taxa per month (Fig. 3A).

Although the total number of species used by honey bees for pollen did not differ across the growing season, more nonnative than native taxa were used during June (June: *F* = 43.81; df = 1, 12; *P* < 0.0001) and July (*F* = 10.91; df = 1, 12; *P* = 0.0063; Fig. 3B, Table 3). There was no difference between representation of native and nonnative pollen types during August (*F* = 1.49; df = 1, 12; *P* = 0.245) and September (*F* = 0.43; df = 1, 12; *P* = 0.5259; Fig. 3B, Table 3).

**Table 3.** Comparisons of taxon richness and percent of pollen derived from two pollen categories (native and nonnative plants) using linear mixed effect models with interaction of pollen category with year or prairie type

Pollen	Month	Effect	df	*F-*value	*P-*value
Taxon richness	June	Pollen category	1, 12	43.81	**<0.0001**
		Prairie type by Pollen category	2, 12	2.08	0.1677
		Year by Pollen category	4, 12	2.8	0.0746
	July	Pollen category	1, 12	10.91	**0.0063**
		Prairie type by Pollen category	2, 12	0.36	0.7064
		Year by Pollen category	4, 12	1.16	0.3768
	Aug.	Pollen category	1, 12	1.49	0.245
		Prairie type by Pollen category	2, 12	6.03	**0.0154**
		Year by Pollen category	4, 12	2.22	0.1282
	Sept.	Pollen category	1, 12	0.43	0.5259
		Prairie type by Pollen category	2, 12	3.47	0.0648
		Year by Pollen category	4, 12	3.58	**0.0382**
Percent	June	Pollen category	1, 12	26.62	**0.0002**
		Prairie type by Pollen category	2, 12	1.09	0.3666
		Year by Pollen category	4, 12	1.06	0.4174
	July	Pollen category	1, 12	9	**0.0111**
		Prairie type by Pollen category	2, 12	0.32	0.733
		Year by Pollen category	4, 12	3.13	0.0559
	Aug.	Pollen category	1, 12	12.72	**0.0039**
		Prairie type by Pollen category	2, 12	1.41	0.2821
		Year by Pollen category	4, 12	0.68	0.6194
	Sept.	Pollen category	1, 12	5.41	**0.0384**
		Prairie type by Pollen category	2, 12	3.16	0.0789
		Year by Pollen category	4, 12	3.77	**0.0327**

Bold values indicate a statistically significant difference.

In the extreme case in which all unidentified taxa were added to the nonnative category, we found no impact on the results for native and nonnative taxon richness comparisons in June, July, and August; but more nonnative than native pollen taxa were collected in September (*F* = 6.15; df = 1, 12; *P* = 0.0289; Supp Table 5 [online only], Supp Fig. 1 [online only]). When all unidentified taxa were added to the native category, there was no significant difference in the amount of pollen collected from native and nonnative plants in July (*F* = 0.08; df = 1, 12; *P* = 0.7804); more native plant taxa were collected by honey bees in August (*F* = 4.99; df = 1, 12; *P* = 0.0454) and September (*F* = 14.18; df = 1, 12; *P* = 0.0027). When we assigned 52 % of the unidentified pollen to nonnative and 48 % to native (percentages reflecting the composition of the identified pollen), the results of this new analysis were unchanged from our initial analysis across all the months (Supp Table 6 [online only]).

### The Amount of Pollen Collected by Honey Bees Varied by Plant Category Throughout the Season

**Fig. 4.**
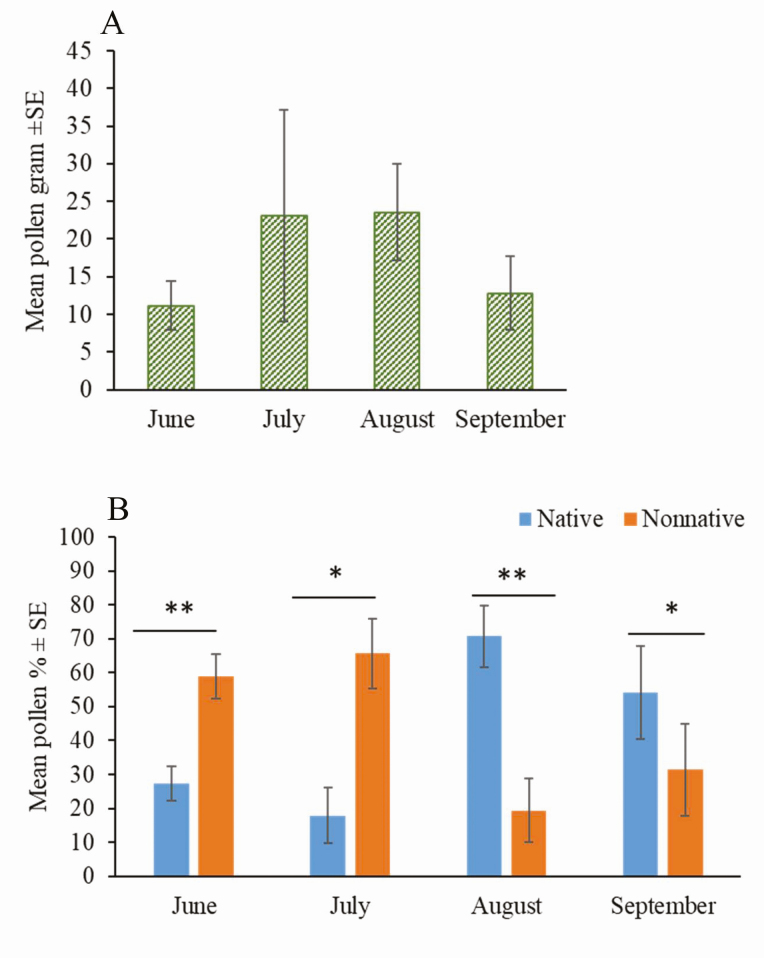
Pollen abundance (g) across the growing season (A) and comparison of percent of pollen by weight derived from native versus nonnative plants (B). A) One pollen collection date in each month was selected for comparing the abundance of pollen across months (see Supp Table 2 [online only] for date selection). Amount of pollen was not significantly different among months (χ ^2^ = 5.35; df = 3; *P* = 0.15). B) Data from all the pollen collection dates were used for comparing percent of pollen derived from native and nonnative plants, and the statistical analysis is summarized in Table 3. We found percent of pollen derived from nonnative plants was significantly higher than that from native plants in June (** *P* < 0.01) and July (* *P* < 0.05), and percent of pollen derived from native plants was significantly higher than that from nonnative plants in August (** *P* < 0.01) and September (* *P* < 0.05).

Prairie-located apiaries collected similar total amounts of pollen across the season (χ ^2^ = 5.35; df = 3; *P* = 0.15; Fig. 4A), but the proportion of native versus nonnative pollen varied seasonally (Fig. 4B). The percent of pollen derived from nonnative plants was significantly greater than that from native plants in June (*F* = 26.62; df = 1, 12; *P* = 0.0002) and July (*F* = 9; df = 1, 12; *P* = 0.0111; Fig. 4B, Table 3). The percent of pollen from native plants was significantly greater than that from nonnative plants in August (*F* = 12.72; df = 1, 12; *P* = 0.0039) and September (*F* = 5.41; df = 1, 12; *P* = 0.0384; Fig. 4B, Table 3).

If all unidentified taxa were added to either nonnative or native categories, we found slightly altered trends in the usage of native plants for pollen (relative to Fig. 4). When all unidentified taxa were assigned to the native category, results for June, August, and September were unaffected; in July, the amount of pollen from native and nonnative plants was not significantly different (*F* = 1.56; df = 1, 12; *P* = 0.2354; Supp Fig. 2 [online only], Supp Table 7 [online only]). When all unidentified taxa were assigned to the nonnative category, results for June, July, and August were unaffected; in September, the amount of pollen from native and nonnative plants was not significantly different (*F* = 1.28; df = 1, 12; *P* = 0.2792). In both of these extreme cases, we observed the same general trends for proportion of native pollen mass collected, consistent with what is reported in Fig. 4; that is, more pollen was derived from nonnative plants in June and more pollen was derived from native plants in August. Only the magnitude of the differences in these months was altered.

### Land Cover and Its Relationship to Collection of Pollen From Native and Nonnative Plants

Overall, grassland cover (included area of prairies where we installed our apiaries) did not explain significant variation in the diversity or abundance of native pollen collected (Table 4). Woodland (*P* = 0.023) and urban land (*P* = 0.0041, Table 4) cover were positively correlated with taxon richness of native plants represented in pollen in September. Urban land and woodland in the surrounding landscapes were positively correlated with percent of native plants represented in pollen in June (*P* = 0.0222) and July (*P* = 0.0180, Table 4), respectively.

**Table 4.** Regression of taxon richness and percent of pollen derived from native plants, considering land cover type surrounding our apiaries within 1.6 km radius

Pollen from native plants^a^	Month	Land cover	Slope	Standard error	*F-*value	*P-*value	Model R^2^
Taxon richness	June	Grassland	0.02719	0.01320	4.24	0.0733	0.3467
	July	Woodland	0.28657	0.1497	3.66	0.0919	0.3142
	Aug.	Urban	−0.11301	0.04921	5.27	0.0507	0.3973
	Sept.	Urban	0.09748	0.03361	8.41	**0.023**	0.5020
		Woodland	0.2536	0.06043	17.61	**0.0041**	0.7738
Percent	June	Urban	2.1965	0.77688	7.99	**0.0222**	0.4998
	July	Woodland	6.68393	2.17626	9.43	**0.0180**	0.5150
		Vacant land	−16.85405	9.91103	2.89	0.1328	0.6568
	Aug.	N/A^b^					
	Sept.	Woodland	9.48501	4.81895	3.87	0.0846	0.3263

Bold values indicate a statistically significant estimation of the parameter.

^a^Percent and taxon richness of pollen were response variables and six general land covers were explanatory variables. ^b^N/A, data did not pass the model selection for exploring a significant relationship.

Overall, the regression analysis supported our prediction that pollen derived from nonnative plants was correlated with nonprairie land cover (such as urban and cropland), with increases in the abundance and taxon richness of nonnative pollen associated with a greater percent of urban or cropland cover in certain months (statistical results summarized in Table 5). For example, in June, both taxon richness of nonnative (*P* = 0.0004, Table 5) and percent of nonnative pollen (*P* = 0.0464, Table 5) collected by honey bees was positively associated with urban land cover. In September, percent of nonnative pollen was positively associated with cropland (*P* = 0.0109, Table 5). Grassland and woodland were negatively associated with diversity of nonnative plants during June (*P* = 0.0274, Table 5) and July (*P* = 0.0421, Table 5), respectively. Wetland cover had a negative association with percent of pollen derived from nonnative plants (*P* = 0.008, Table 5).

**Table 5.** Regression of taxon richness and percent of pollen derived from nonnative plants, considering land cover type surrounding our apiaries within 1.6 km radius

Pollen from nonnative plants^a^	Month	Land cover	Slope	Standard error	*F-*value	*P-*value	R^2^
Taxon richness	June	Urban	0.15233	0.02447	38.76	**0.0004**	0.7502
		Grassland	−0.01871	0.00674	7.71	**0.0274**	0.8811
	July	Woodland	−0.28665	0.11867	5.83	**0.0421**	0.4217
	Aug.	N/A^b^					
	Sept.	Wetland	0.11613	0.05302	4.80	0.0599	0.3749
Percent	June	Urban	3.43787	1.42303	5.84	**0.0464**	0.2933
		Woodland	4.09145	2.48372	2.71	0.1435	0.4907
	July	Wetland	−4.64442	1.32319	12.32	**0.008**	0.6063
	Aug.	Grassland	−0.89201	0.52412	2.90	0.1272	0.2658
	Sept.	Cropland	1.44687	0.43876	10.87	**0.0109**	0.5761

Bold values indicate a statistically significant estimation of the parameter.

^a^Percent and taxon richness of pollen were response variables and six general land covers were explanatory variables. ^b^N/A, data did not pass the model selection for exploring a significant relationship.

## Discussion

Honey bees are a globally-distributed, semidomesticated insect with highly polyphagous feeding habits (Kaluza et al. 2017). Although honey bees are known to forage on a wide variety of crops, weeds, as well as native species in many regions (Sponsler et al. 2017), the extent to which they use native versus nonnative plants outside of their original range is not well-understood. We provide a detailed assessment of how honey bees used native and nonnative plants for pollen across the growing season, in the context of reconstructed tallgrass prairies in the Midwestern United States, a critical area for pollinator conservation as well as bee health (Grixti et al. 2009, Zaya et al. 2017). Our results suggest honey bees use native plants in prairies throughout the season, even though honey bees did not coevolve with native Midwestern United States prairie plants. This finding confirms that honey bees as generalist foragers can adapt to versatile habitats within their introduced range. Honey bees utilized more nonnative plants earlier in the season but used more native prairie plants later in the season. This suggests that native habitats may provide an especially important source of pollen to honey bees later in the season, a time of forage dearth observed in central Iowa (Dolezal et al. 2019).

Overall, 12 native plant taxa that occur in Iowa prairies were identified in the pollen collected by honey bees. Our visual inspection of flowering plants found adjacent to colonies revealed the presence of nine of those taxa (except *Oenothera biennis* L. [Myrtales: Onagraceae], *Sambucus Canadensis* L. [Dipsacales: Adoxaceae] and *Tilia Americana* L. [Malvales: Malvaceae]), suggesting that honey bees utilized prairies for pollen. Pollen from native prairie plants was found in pollen traps across the entire season, with different species represented at varying times. For example, northern blue flag (*I. versicolor*) and purple prairie clover (*D. purpurea*) were collected during June and July, and partridge pea (*C. fasciculata*), goldenrod (*Solidago* spp.), and sunflowers (*Helianthus* spp.) were collected during August and September. These time-periods overlap with the flowering phenology of these taxa (Henry 2002; USDA-NRCS 2002, 2003; Carr 2009; Pavek 2011; Houck and Row 2019). Honey bees are likely to use prairie plants depending upon both their flowering phenology and the flowering of nonnative species.

Honey bees frequently used nonnative plants throughout the season. Honey bees may have found these nonnative plants within the prairies, but more likely they were found in landscape features such as crop fields, field margins, and roadsides. For example, a significant amount of pollen from corn was found in traps during July of 2018, likely from surrounding corn fields that were in anthesis. The taxon richness and abundance of pollen in relation to variation in the surrounding landscape suggests that urban and crop landscapes may be a source of pollen derived from nonnative plants. The pattern of using both native and nonnative plants as a source of pollen suggests honey bees are modifying their foraging behavior based on the availability of flowering of plants within different features of their overall foraging landscape. Our observations and analyses were consistent with a recent study that analyzed the dance language of honey bee foragers and compositions of pollen collected by them that indicate a simultaneous use of prairies and other land covers in Midwestern U.S. landscapes (Carr-Markell et al. 2020).

The balance of pollen from native or nonnative plants varied significantly by month. During June and July, honey bees collected more pollen from nonnative plants than native plants; while, during August and September, more pollen was collected from native plants. This pattern suggests that as nonnative plants stop blooming, honey bees switch to native plants blooming later in the growing season. The early predominance of nonnative plants represented in bee-collected pollen may be due to greater attraction or availability of pollen from nonnative plants. Both honey bees and some nonnative plants originated from Europe, such as white clover, red clover, and sweet clover, and may have a coevolutionary history such that honey bees continue to prefer these plants when both are found in North America. For example, the length of the flower tubes for these plants is shorter or equal to the extended proboscis of honey bees, making it easier for foragers to reach the nectar or pollen in the flower (Alexandersson and Johnson 2002). Foraging preference on those nonnative plants could be related to high nutritional value of their pollen (Rayner and Langridge 1985, Russo et al. 2019). In contrast, honey bees may have a low preference for some native plants, for example, *Ratibida pinnata* is an abundant native prairie plant, observed frequently at locations used for this study early in the season, but was not found in the pollen traps. Greater amount of pollen from nonnative plants can also be explained by plant availability and blooming time. Many nonnative plants that successfully colonize outside their native habitats tend to flourish in disturbed habitats such as field edges of croplands and roadsides in urban lands by taking advantages of an ecological niche and have an adaption strategy of blooming early for a successful reproduction (Grime 2006, Colautti and Barrett 2013). For example, nonnative clover species such as white clover (*Trifolium repens*) are commonly found in field edges and roadsides in this study region and start blooming during the early part of the growing season. As noted by Dolezal et al. (2019), clover bloom declines in August in central Iowa, which may facilitate a switch to more abundant native plants found in prairies. Plant surveys conducted in the prairies used in this study revealed a diverse community of native plants that flower throughout a growing season, including August and September (Shepherd and Debinski 2005, Ohnesorg 2008, Orlofske et al. 2011, Summerville et al. 2011, Delaney et al. 2015).

We were unable to survey the entire potential foraging area of each apiary (e.g., 314 km^2^ if honey bees scout up to a 10 km radius) for all the flowering plants that could be used for pollen. This resulted in some plants not being included in our reference library for identifying pollen through microscopy, with unidentified pollen representing an average of 15 % of the total pollen collected in each month. We performed a series of analyses to assess robustness of our findings when hypothetically assigning unidentified pollen taxa to native versus nonnative groups. Overall, these analyses suggest our inability to identify the source of pollen for all the plants represented in our collection could have some minor impacts on the results (e.g., the finding that native taxa were more abundant in August depended on the group assignment of unidentified species). However, none of the hypothetical re-analyses significantly changed our finding for balance of native versus nonnative species across seasons. In other words, our finding that honey bees used more nonnative plants earlier in the season and more native plants later in the season appears to be quite robust.

Prairie plants may provide a source of nectar for honey bees in this landscape after other common plants in the Midwest cease blooming. Honey bees kept adjacent to commercial soybean fields in central Iowa suffered colony weight loss beginning in August (Dolezal et al. 2019). Colony weight peaked in August, followed by a steep decline that appeared to coincide with forage dearth. This weight loss was reversed by giving honey bees access to reconstructed prairies (Dolezal et al. 2019), which contain numerous native plants that flower after August and may provide important sources of late-season nectar (e.g., goldenrod). Extending the results of Dolezal et al. (2019), our results suggest that access to prairies could also help honey bees avoid a shortage of pollen later in the season. Late-season pollen may provide an important source of protein and lipids that can enhance fat body growth for ‘winter bee’ workers that will need extra nutrient stores to survive the winter (Döke et al. 2015). Improvements to the abundance and diversity of pollen consumed by honey bees can result in improved survival of adult honey bees when exposed to viral pathogens (Dolezal et al. 2019, Zhang et al. 2020). Future experiments should consider if the pollen derived from prairie plants directly benefit the health of honey bees.

In addition to studying the pattern of pollen collection across seasons, we also estimated the impact of prairie type (integrated versus isolated prairies). We found pollen collected by colonies in integrated tallgrass prairie was more abundant than that collected in isolated tallgrass prairies (Supp Table 8 [online only]). This may be due to colonies located in integrated tallgrass prairies having greater access to native forage than these placed in isolated prairies, first, because of larger areas of each integrated prairie, and second, because of adjacency to other surrounding prairies. In contrast, colonies kept in isolated tallgrass prairies may lack sources of native forage in the surrounding landscapes. Future studies should consider aspects of the foraging response of honey bees to the varying size of prairies. We did not survey the prairie plant community and the future studies should consider honey bees’ response to plant community of different compositions in prairies.

In conclusion, we observed that when apiaries were placed in prairies in central Iowa, honey bees used many members of the plant community that are native to North American prairies. Although cultivated areas can be an important source of pollen in June and July (especially nonnative species such as clover), prairies became a more important source of pollen in August and September. The native plants such as partridge pea and goldenrod could buffer late-season colony decline when floral resources in cultivated areas have declined steeply. Because of access to diverse plants in prairies, colonies did not suffer from a shortage of pollen later in the season. If a habitat is created to benefit honey bees, increasing the diversity of native plants that are used as a source of forage by honey bees should be considered. It has been suggested that conservation for honey bees focuses on a simpler seed mix primarily composed of two nonnative species (*Melilotus* and *Medicago*) and one native species (*Linum*) (Otto et al. 2017). Although these plants may be preferred by honey bees, they mainly flower in the early part of the growing season. Our results suggest that honey bees’ use of native plants may depend upon the seasonality of both native and nonnative plants present in the landscape. Planting a more diverse mixture of forbs, especially in regions that experience precipitous declines in floral resources, can support honey bees as well as wild pollinators. If the goal is to benefit both, it may be possible to limit potential competition by selecting a mixture of plants that contain preferred sources for honey bees, as well as some species that are more preferred by wild pollinators. Overall, these results suggest native prairie restoration may be a conservation management strategy that can provide benefits to managed honey bees, while also benefiting native biodiversity.

## Supplementary Material

ieab001_suppl_Supplementary_Materials
