# Supplementary material for: North American Prairie Is a Source of Pollen for Managed Honey Bees (Hymenoptera: Apidae)
Source: J Insect Sci. 2021 Feb 23;21(1):17. doi: 10.1093/jisesa/ieab001 (PMC7901588; doi:10.1093/jisesa/ieab001)
Supplement: ieab001_suppl_Supplementary_Materials [file ieab001_suppl_supplementary_materials.docx]

**Supplementary materials**

Supp. Table 1. The land covers within 1.6 km radius of each apiary grouped into six major types.

| Six major types | Detailed land cover |
| --- | --- |
| 1. Cropland | Corn, soybean, sweet corn, winter wheat, rye, oats, apple orchard |
| 2. Urban | Developed/open space, developed/low intensity, developed/medium intensity, developed/high intensity |
| 3. Grassland | Alfalfa, non alfalfa hay, sod/grass seed, fallow, grass/pasture |
| 4. Woodland | Deciduous forest, evergreen forest, shrubland |
| 5. Wetland | Woody wetlands, herbaceous wetlands |
| 6. Vacant-land | Barren land, open water |

Supp. Table 2. Pollen collection frequency in each month of the study across three years.

| Month | 2016 | 2017 | 2018 |
| --- | --- | --- | --- |
| June | 5 (3rd, 8th 16th, 23rd, **29th**) * | 1 (**26th**) | 2 (13th, **28th**) |
| July | 4 (9th,15th, 23rd, **27th**) | 2 (7th, **25th**) | 2 (11th, **27th**) |
| August | 3 (4th, 10th, **18th**) | 2 (4th, **23rd**) | 2 (11th, **28th**) |
| September | 1 (**4th**) | 3 (**5th**, 16th, 25th) | 1 (**7th**) |

* Total number of collection (the exact collection date) within a month. The dates in bold were selected for determining the variation in diversity and abundance across months.

Supp. Table 3. Plant species included in our reference pollen library that were collected in Iowa during 2015-2018.

| Family name | Scientific name | Common name | Plant type | Nativeness ^a^ | Invasiveness ^b^ |
| --- | --- | --- | --- | --- | --- |
| Amaranthaceae | *Amaranthus tuberculatus* | Common waterhemp | Herb | Native | Invasive |
| Asteraceae | *Ambrosia artemisiifolia* | Common ragweed | Herb | Native | Invasive |
| Asteraceae | *Ambrosia trifida* | Giant ragweed | Herb | Native | Invasive |
| Convolvulaceae | *Ipomoea nil* | Japanese morning glory | Herb | Native | Invasive |
| Brassicaceae | *Lepidium virginicum* | Virginia pepperweed | Herb | Native | Invasive |
| Cucurbitaceae | *Sicyos angulatus* | Bur cucumber | Herb | Native | Invasive |
| Asteraceae | *Achillea millefolium* | Yarrow | Herb | Native | Noninvasive |
| Asteraceae | *Ageratina altissima* | White snakeroot | Herb | Native | Noninvasive |
| Fabaceae | *Amorpha canescens* | Lead plant | Herb | Native | Noninvasive |
| Ranunculaceae | *Anemone canadensis* | canada anemone | Herb | Native | Noninvasive |
| Fabaceae | *Baptisia alba* | White wild indigo | Herb | Native | Noninvasive |
| Bignoniaceae | *Catalpa speciosa* | Northern catalpa | Tree or shrub | Native | Noninvasive |
| Fabaceae | *Chamaecrista fasciculata* | Partridge pea | Herb | Native | Noninvasive |
| Santalaceae | *Comandra umbellata* | Bastard toadflax | Herb | Native | Noninvasive |
| Asteraceae | *Coreopsis palmata* | Prairie coreopsis | Herb | Native | Noninvasive |
| Cucurbitaceae | *Cucurbita pepo* | Pumpkin | Herb | Native | Noninvasive |
| Fabaceae | *Dalea candida* | White prairie clover | Herb | Native | Noninvasive |
| Fabaceae | *Dalea purpurea* | Purple prairie clover | Herb | Native | Noninvasive |
| Fabaceae | *Desmanthus illinoensis* | Prairie minosa | Herb | Native | Noninvasive |
| Fabaceae | *Desmodium canadense* | Showy tick-trefoil | Herb | Native | Noninvasive |
| Rosaceae | *Drymocallis arguta* | Prairie cinquefoil | Herb | Native | Noninvasive |
| Asteraceae | *Echinacea pallida,* | Pale purple cone flower | Herb | Native | Noninvasive |
| Asteraceae | *Echinacea purpurea* | Purple coneflower | Herb | Native | Noninvasive |
| Asteraceae | *Erigeron strigosus* | Prairie fleabane | Herb | Native | Noninvasive |
| Apiaceae | *Eryngium yuccifolium* | Rattle snake master | Herb | Native | Noninvasive |
| Rosaceae | *Fragaria vesca* | Wild strawberry | Herb | Native | Noninvasive |
| Asteraceae | *Helianthus annuus* | Common sunflower | Herb | Native | Noninvasive |
| Asteraceae | *Helianthus decapetalus* | Thinleaf sunflower | Herb | Native | Noninvasive |
| Asteraceae | *Helianthus grosserratus* | Sawtooth sunflower | Herb | Native | Noninvasive |
| Asteraceae | *Heliopsis helianthoides* | False sunflower | Herb | Native | Noninvasive |
| Iridaceae | *Iris versicolor* | Blue flag | Herb | Native | Noninvasive |
| Fabaceae | *Lespedeza capitata* | Round-headed bush clover | Herb | Native | Noninvasive |
| Lamiaceae | *Monarda fistulosa* | Wild bergomot | Herb | Native | Noninvasive |
| Onagraceae | *Oenothera biennis* | Evening primrose | Herb | Native | Noninvasive |
| Asteraceae | *Penstemon digitalis* | Foxglove beard tongue | Herb | Native | Noninvasive |
| Polemoniaceae | *Phlox paniculata* | Garden phlox | Herb | Native | Noninvasive |
| Lamiaceae | *Pycnanthemum virginianum* | Virginia mountain mint | Herb | Native | Noninvasive |
| Asteraceae | *Ratibida pinnata* | Prairie coneflower | Herb | Native | Noninvasive |
| Rosaceae | *Rosa blanda* | Prairie rose | Tree or shrub | Native | Noninvasive |
| Asteraceae | *Rudbeckia hirta* | Black-eyed susan | Herb | Native | Noninvasive |
| Adoxaceae | *Sambucus spp.* | Elderberry | Tree or shrub | Native | Noninvasive |
| Asteraceae | *Silphium integrifolium* | Rosinweed | Herb | Native | Noninvasive |
| Asteraceae | *Silphium laciniatum* | Compass plant | Herb | Native | Noninvasive |
| Asteraceae | *Silphium perfoliatum* | Cup plant | Herb | Native | Noninvasive |
| Asteraceae | *Solidago canadensis* | Canada goldenrod | Herb | Native | Noninvasive |
| Asteraceae | *Solidago rigida* | Stiff goldenrod | Herb | Native | Noninvasive |
| Asteraceae | *Solidago speciosa* | Showy goldenrod | Herb | Native | Noninvasive |
| Asteraceae | *Symphyotrichum ericoides* | White heath aster | Herb | Native | Noninvasive |
| Malvaceae | *Tilia americana* | Basswood | Tree or shrub | Native | Noninvasive |
| Commelinaceae | *Tradescantia virginiana* | Spider wort | Herb | Native | Noninvasive |
| Lamiales | *Verbena stricta* | Hoary vervain | Herb | Native | Noninvasive |
| Asteraceae | *Vernonia noveboracensis* | Iron weed | Herb | Native | Noninvasive |
| Violaceae | *Viola papilionacea* | Wild violet | Herb | Native | Noninvasive |
| Apiaceae | *Zizia aurea* | Golden Alexander | Herb | Native | Noninvasive |
| Malvaceae | *Abutilon theophrasti* | Velvetweed | Herb | Nonnative | Invasive |
| Asteraceae | *Carduus nutans* | Musk thistle | Herb | Nonnative | Invasive |
| Amaranthaceae | *Chenopodium album* | Common lambsquarters/pigweed | Herb | Nonnative | Invasive |
| Asteraceae | *Cirsium arvense* | Canada thistle | Herb | Nonnative | Invasive |
| Asteraceae | *Cirsium vulgare* | Bull thistle | Herb | Nonnative | Invasive |
| Convolvulaceae | *Convolvulus arvensis* | Field bindweed | Herb | Nonnative | Invasive |
| Apiaceae | *Daucus carota* | Queen Anne's lace | Herb | Nonnative | Invasive |
| Fabaceae | *Melilotus albus* | White sweet clover | Herb | Nonnative | Invasive |
| Fabaceae | *Melilotus officinalis* | Yellow sweet clover | Herb | Nonnative | Invasive |
| Apiaceae | *Pastinaca sativa* | Wild parsnip | Herb | Nonnative | Invasive |
| Polygonaceae | *Persicaria maculosa* | Redshank | Herb | Nonnative | Invasive |
| Solanaceae | *Physalis peruviana* | Cape gooseberry | Herb | Nonnative | Invasive |
| Plantaginaceae | *Plantago lanceolata* | Ribwort plantain | Herb | Nonnative | Invasive |
| Caryophyllaceae | *Saponaria officinalis* | Bouncing bet | Herb | Nonnative | Invasive |
| Caryophyllaceae | *Silene latifolia* | White campion | Herb | Nonnative | Invasive |
| Asteraceae | *Sonchus arvensis* | Field sow thistle | Herb | Nonnative | Invasive |
| Asteraceae | *Taraxacum officinale* | Common dandelion | Herb | Nonnative | Invasive |
| Asteraceae | *Tragopogon dubius* | Yellow salsify | Herb | Nonnative | Invasive |
| Asparagaceae | *Asparagus officinalis* | Asparagus | Herb | Nonnative | Noninvasive |
| Brassicaceae | *Brassica napus* | Rapaseed | Herb | Nonnative | Noninvasive |
| Asteraceae | *Cichorium intybus* | Common chicory | Herb | Nonnative | Noninvasive |
| Fabaceae | *Glycine max* | Soybean | Herb | Nonnative | Noninvasive |
| Liliaceae | *Lilium lancifolium* | Tiger lily | Herb | Nonnative | Noninvasive |
| Fabaceae | *Medicago sativa* | Alfalfa | Herb | Nonnative | Noninvasive |
| Apiaceae | *Myrrhis odorata* | Sweet cicely | Herb | Nonnative | Noninvasive |
| Papaveraceae | *Papaver somniferum* | Opium poppy | Herb | Nonnative | Noninvasive |
| Fabaceae | *Phaseolus vulgaris* | Green bean | Herb | Nonnative | Noninvasive |
| Fabaceae | *Securigera varia* | Crown vetch | Herb | Nonnative | Noninvasive |
| Fabaceae | *Trifolium hybridum* | Alsike clover | Herb | Nonnative | Noninvasive |
| Fabaceae | *Trifolium incarnatum* | Crimson clover | Herb | Nonnative | Noninvasive |
| Fabaceae | *Trifolium pratense* | Red clover | Herb | Nonnative | Noninvasive |
| Fabaceae | *Trifolium repens* | White clover | Herb | Nonnative | Noninvasive |
| Scrophulariaceae | *Verbascum thapsus* | Common mullein | Herb | Nonnative | Noninvasive |
| Poaceae | *Zea may* | Corn | Herb | Nonnative | Noninvasive |
| Salicaceae | *Salix* spp. | Willow | Tree or shrub | Unknown | Noninvasive |

^a^ Nativeness and ^b^ invasiveness of each plant was decided based on the information in Plants Database of Natural Resources Conservation Service of United States Department of Agriculture <https://plants.sc.egov.usda.gov/java/>.

Supp. Table 4. Mean percent of pollen that was unidentified in our study.

| Unidentified  taxa | 2016 (Mean ± SE, n = 5) | | | | 2017 (Mean ± SE, n = 2) | | | | 2018(Mean ± SE, n = 3) | | | |
| --- | --- | --- | --- | --- | --- | --- | --- | --- | --- | --- | --- | --- |
|  | June | July | August | September | June | July | August | September | June | July | August | Sept |
| UIPT1* | 0.08±0.05 | 0.02±0.02 | 0.13±0.13 | 0 | 0 | 45.7±44.99 | 0.46±0.46 | 0.67±0.67 | 0.44±0.41 | 0 | 1.17±0.78 | 0 |
| UIPT2 | 0 | 0 | 0.02±0.01 | 0 | 0 | 0 | 0 | 0 | 0 | 0 | 0 | 0 |
| UIPT3 | 0.01±0.01 | 0 | 0 | 0 | 0 | 0 | 0 | 0 | 0 | 0 | 0 | 0 |
| UIPT4 | 0.03±0.03 | 0 | 0 | 0 | 0 | 0 | 0 | 0 | 0 | 0 | 0 | 0 |
| UIPT5 | 11.09±4.8 | 0.01±0.01 | 0.08±0.08 | 0 | 0 | 0 | 0 | 0 | 0 | 0 | 0 | 0 |
| UIPT6 | 0.6±0.59 | 0 | 0 | 0 | 0 | 0 | 0 | 0 | 0 | 0 | 0 | 0 |
| UIPT7 | 7.82±4.52 | 0 | 0 | 0 | 0 | 0 | 0 | 0 | 0 | 0 | 0 | 0 |
| UIPT8 | 0.11±0.07 | 0 | 0 | 0 | 0 | 0 | 0 | 0 | 0 | 0 | 0 | 0 |
| UIPT9 | 0.01±0.01 | 0 | 0 | 0 | 0 | 0 | 0 | 0 | 0 | 0 | 0 | 0 |
| UIPT10 | 0 | 0.67±0.67 | 0 | 0 | 0 | 0 | 0 | 0 | 0 | 0 | 0 | 0 |
| UIPT11 | 0 | 0.01±0.01 | 0.59±0.59 | 2.75±1.22 | 0 | 0 | 0 | 0 | 0 | 0 | 0 | 0 |
| UIPT12 | 0 | 0.26±0.25 | 0.03±0.03 | 0 | 0 | 0 | 0 | 0 | 0 | 0 | 0 | 0 |
| UIPT13 | 0.8±0.58 | 0.04±0.04 | 0 | 0 | 0 | 0 | 0 | 0 | 0 | 0 | 0 | 0 |
| UIPT14 | 0 | 0 | 0 | 0.18±0.18 | 0 | 0 | 0 | 0.02±0.02 | 0 | 0 | 21.07±8.4 | 0 |
| UIPT15 | 0.24±0.15 | 0 | 0.02±0.02 | 0 | 0 | 0 | 0 | 0 | 0 | 0 | 0 | 0 |
| UIPT16 | 1.82±1.35 | 0 | 0 | 0 | 0 | 0 | 0 | 0 | 0 | 0 | 0 | 0 |
| UIPT17 | 0 | 0 | 0.02±0.02 | 0 | 0 | 0 | 0 | 0 | 0 | 0 | 0 | 0 |
| UIPT18 | 0.3±0.11 | 0 | 0 | 0 | 0 | 0.35±0.35 | 0 | 0 | 4.01±2.09 | 0.6±0.32 | 0 | 0 |
| UIPT19 | 0 | 0 | 0 | 0 | 0 | 0 | 0 | 0.03±0.03 | 0 | 0 | 0 | 0 |
| UIPT20 | 0 | 0 | 0 | 0 | 0 | 0 | 0 | 1.61±1.61 | 0 | 0 | 0 | 0 |
| UIPT21 | 0 | 0 | 0 | 0 | 0 | 0 | 0 | 0 | 0 | 0.05±0.05 | 0 | 0 |
| UIPT22 | 0 | 0 | 0 | 0 | 0 | 0 | 0 | 0 | 1.32±1.32 | 0 | 0 | 0 |
| UIPT23 | 0 | 0 | 0 | 0 | 0 | 0 | 0 | 0 | 0.13±0.13 | 0.04±0.04 | 0.19±0.19 | 0 |
| UIPT24 | 0 | 0 | 0 | 0 | 0 | 0 | 0 | 0 | 0.31±0.18 | 0 | 0 | 0 |
| UIPT25 | 0 | 0 | 0 | 0 | 0 | 0 | 0 | 0 | 0.66±0.66 | 0 | 0 | 0 |
| UIPT26 | 0 | 0 | 0 | 0 | 0 | 0 | 0 | 0 | 0.04±0.04 | 0 | 0 | 0 |
| UIPT27 | 0 | 0 | 0 | 0 | 0 | 0 | 0 | 0 | 0.65±0.42 | 0 | 0 | 0 |
| UIPT28 | 0 | 0 | 0 | 0 | 0 | 0 | 0 | 0 | 0 | 0 | 5.52±1.89 | 1.12±1.12 |
| UIPT29 | 0 | 0 | 0 | 0 | 0 | 0 | 0 | 0 | 0 | 18.03±16.6 | 0 | 23.55±23.55 |
| UIPT30 | 0 | 0 | 0 | 0 | 0 | 0 | 0 | 0 | 0 | 1.51±1.51 | 0 | 0 |
| UIPT31 | 0 | 0 | 0 | 0 | 0 | 0 | 0 | 0 | 0 | 0.26±0.15 | 0 | 0 |
| UIPT32 | 0 | 0 | 0 | 0 | 0 | 0 | 0 | 0 | 0 | 1.97±1.94 | 3.21±2.63 | 17.08±4.14 |

* UIPT, unidentified pollen taxa.

Supp. Table 5. Comparisons of taxon richness of native and nonnative plant categories with all the unidentified taxa added to either native or nonnative, using the same linear mixed models as Table 3 in the main manuscript.

| Unidentified taxa | Month | df | F value | P value |
| --- | --- | --- | --- | --- |
| Added to native | June | 1, 12 | 9.17 | 0.0105 |
|  | July | 1, 12 | 0.08 | 0.7804 |
|  | August | 1, 12 | 4.99 | 0.0454 |
|  | September | 1, 12 | 14.18 | 0.0027 |
| Added to nonnative | June | 1, 12 | 63.4 | <.0001 |
|  | July | 1, 12 | 25.09 | 0.0003 |
|  | August | 1, 12 | 25.72 | 0.0003 |
|  | September | 1, 12 | 6.15 | 0.0289 |

Supp. Table 6 Comparisons of taxon richness of native and nonnative plant categories with 50 % of unidentified taxa added to native categories, using the same linear mixed models as Table 3 in the main manuscript.

| Month | df | F Value | P value |
| --- | --- | --- | --- |
| June | 1, 12 | 21.48 | 0.0006 |
| July | 1, 12 | 8.47 | 0.0131 |
| August | 1, 12 | 3.13 | 0.1025 |
| September | 1, 12 | 0.15 | 0.7066 |

Supp. Table 7. Comparisons of percent of pollen derived from native and nonnative plant categories with unidentified pollen added to either native or nonnative, using the linear mixed models same to Table 3 in the main manuscript.

| Unidentified taxa | September | df | F value | P value |
| --- | --- | --- | --- | --- |
| Added to native | June | 1, 12 | 15.42 | 0.002 |
|  | July | 1, 12 | 1.56 | 0.2354 |
|  | August | 1, 12 | 19.04 | 0.0009 |
|  | September | 1, 12 | 15.13 | 0.0022 |
| Added to nonnative | June | 1, 12 | 40.33 | <.0001 |
|  | July | 1, 12 | 18.6 | 0.001 |
|  | August | 1, 12 | 7.8 | 0.0162 |
|  | September | 1, 12 | 1.28 | 0.2792 |

Supp. Table 8. Comparison of taxon richness of plants as pollen source and pollen abundance between prairie type (ISO versus INT) using the linear mixed models.

| Pollen | Effect | df | F value | P value |
| --- | --- | --- | --- | --- |
| Taxon richness | Prairie type | 1, 36 | 4.82 | 0.0346 ^a^ |
|  | Year × Prairie type | 2, 36 | 4.87 | 0.0135 |
| Percent | Prairie type | 1, 36 | 7.87 | 0.0081 ^b^ |
|  | Year × Prairie type | 2, 36 | 0.32 | 0.7309 |

^a^ The difference of least square means of pollen from INT and ISO through the linear mixed model indicates a higher richness from INT (estimate = 1.2083, SE = 0.5503; t = 2.2; df = 36; *P* = 0.0346).

^b^ The difference of least square means of pollen from INT and ISO through the linear mixed model indicates a higher richness from INT (estimate = 1.1659, SE = 0.4157; t = 2.80; df = 36; *P* = 0.0081).

A


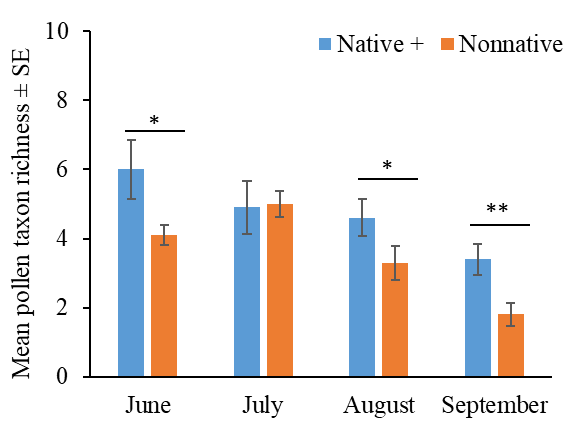

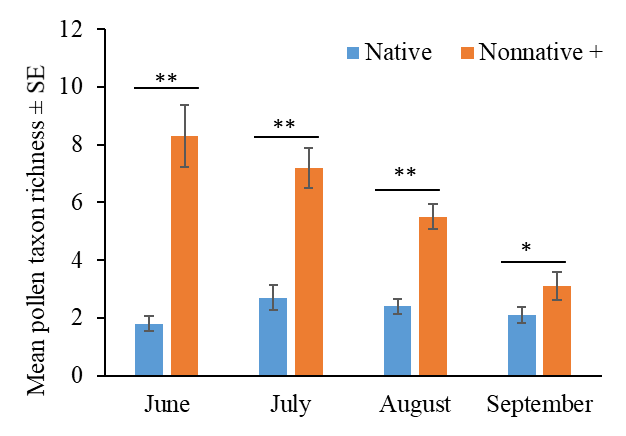


B

Supp. Figure 1. Comparisons of taxon richness of pollen derived from native and nonnative plants with all the unidentified pollen taxa added to native (A) or nonnative category (B). The cross symbol (+) behind “Native” in plot A and “Nonnative” in plot B indicates the category of pollen added with unidentified pollen. Refer to Supp. Table 5 for statistical results. ** *P* < 0.01 and * *P* < 0.05.

A


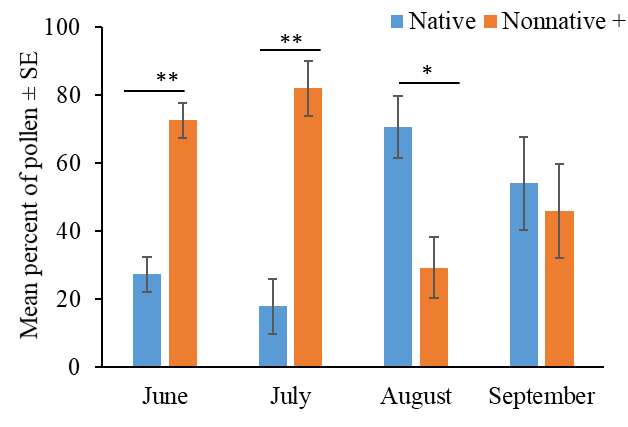


B


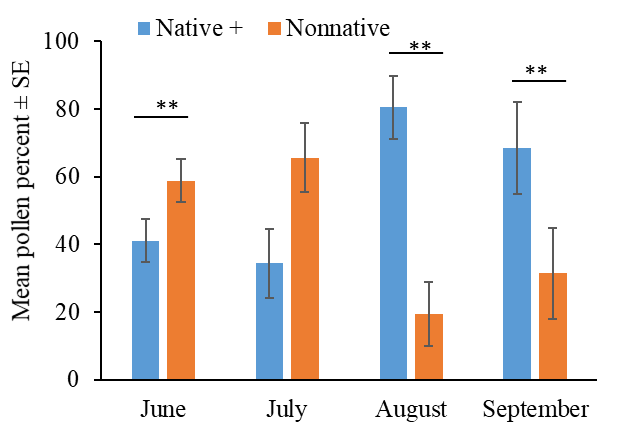


Supplemental Figure 2. Comparisons of percent of pollen derived from native and nonnative plants with all the unidentified pollen taxa added to nonnative category (A) and native (B). The cross symbol (+) behind “Native” in plot A and “Nonnative” in plot B indicates the category of pollen added with unidentified pollen. Refer to Supp. Table 7 for statistical results. ** *P* < 0.01 and * *P* < 0.05.
